# Nuclear Structure Studies With the Inelastic Neutron Scattering Reaction and Gamma-Ray Detection

**DOI:** 10.6028/jres.105.019

**Published:** 2000-02-01

**Authors:** P. E. Garrett, N. Warr, S. W. Yates

**Affiliations:** Lawrence Livermore National Laboratory Livermore, CA 94551; University of Kentucky Lexington, KY 40506-0055

**Keywords:** Doppler-shift attenuation method, γγ-coincidences, inelastic neutron scattering, nuclear structure

## Abstract

The (n,n′γ) reaction has been used at the University of Kentucky accelerator facility to examine the detailed structure of a number of nuclei. The advantages of this method are reviewed, and recent developments are described. Examples of unique nuclear structure studies that have been carried out with this method are presented.

## 1. Introduction

The inelastic neutron scattering reaction [1,2] with γ-ray detection has been used for many years in studies of the nuclear structure of stable nuclei. This reaction affords several advantages over other methods.

- With monoenergetic, accelerator-produced neutrons (and no Coulomb barrier), levels can be examined close to the threshold for their excitation without the attendant complications associated with radiation from higher-lying levels.
- At low incident-neutron energies, this reaction is generally non-selective. The angular momentum that can be brought into the system by low-energy neutrons is a limitation, but this is generally not a serious deficiency in low-spin studies.
- The energy resolution for γ ray detection is good and typically much better than that for detecting other forms of nuclear radiation.

On the other hand, the (n,n′ γ) reaction is practically limited to stable nuclei and large amounts of material, frequently enriched isotopic samples, are required.

At the University of Kentucky accelerator laboratory, we have employed the inelastic neutron scattering (INS) reaction, i.e., (n,n′ γ), for many years in nuclear structure studies. The methods developed, as well as recent innovations, are presented.

## 2. Neutron Production and Gamma-Ray Detection

Neutrons are produced at the University of Kentucky accelerator using the reactions

$${}^{1}H{+}^{3}H\to^{3}\mathrm{He}+n\;Q=-0.764\;\mathrm{MeV}$$ (1)

$${}^{2}H{+}^{2}H\to^{3}\mathrm{He}+n\;Q=3.269\;\mathrm{MeV}$$ (2)

with gaseous targets. As the accelerator, a Model CN Van de Graaff, typically operates at voltages below 6.5 MV, essentially monoenergetic neutrons with continuously variable energies up to 9.5 MeV are available. Gamma rays produced in the (n,n′γ) reaction are detected with high-purity germanium (HPGe) semiconductor detectors.

## 3. Excitation Functions and Angular Distribution Measurements

**Fig. 1:**
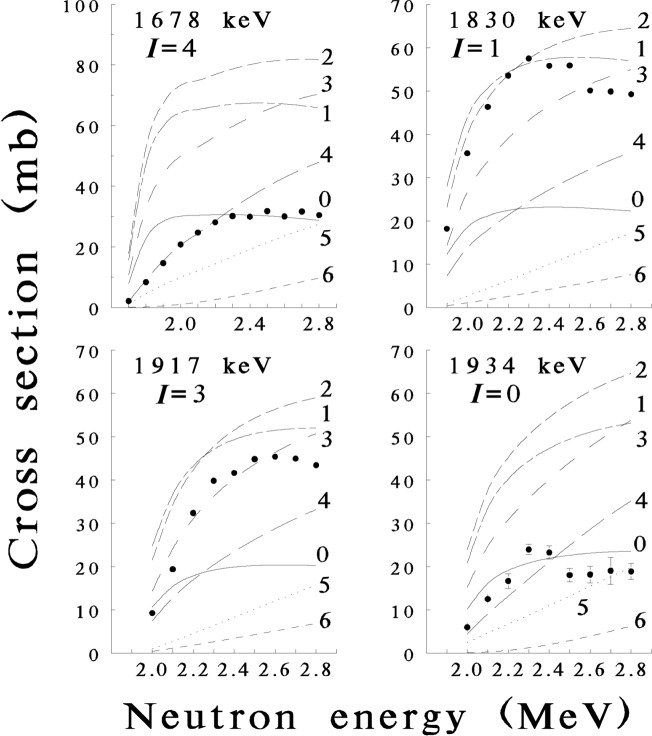
Excitation function yields extracted for ^166^Er. The points (with error bars where larger than the dots) are compared with CINDY [3] calculations (lines) for various spins. The level energy and spin are indicated. Differences in predicted cross sections due to parity are small; only those results for the known parity of the level are shown. The disagreement between data and calculation above 2.4 MeV is attributed to the choice of optical model parameters and incomplete knowledge of discrete levels.

The accurate determination of the yield threshold for a particular γ ray is used to place uniquely the level from which the transition arises, and these excitation functions of γ-ray yields (cross sections) are also useful for inferring level spins and parities. Examples of yield and threshold determinations, obtained from the ^166^Er(n,n′ γ) reaction, are shown in Fig. 1.

**Fig. 2:**
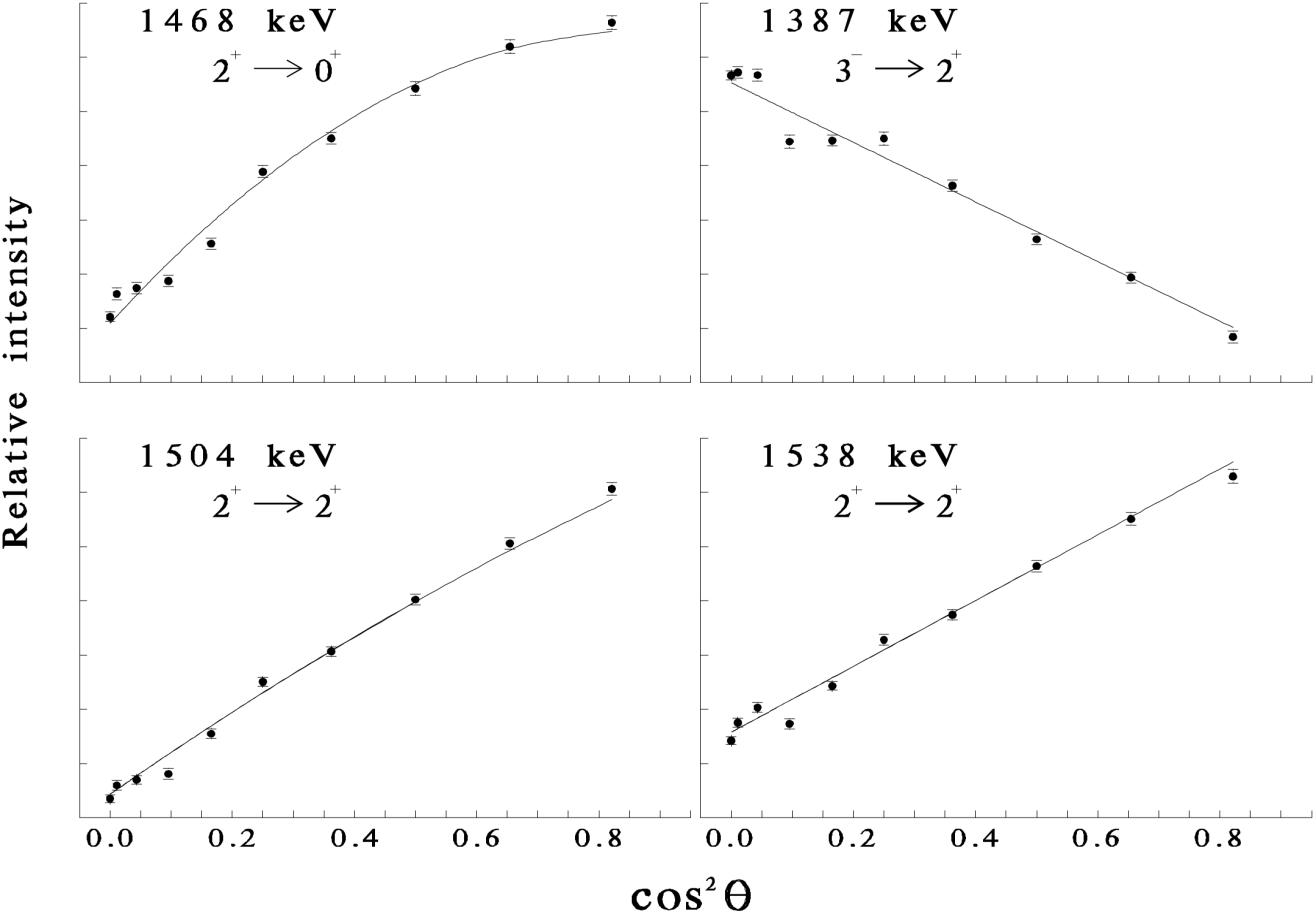
Angular distributions for selected transitions in ^112^Cd. The data are compared with CINDY [3] calculations (line) for the transition indicated. For cases where mixed E2/M1 transitions are possible, the line shown is calculated for the value of δ which minimizes the *χ*^2^.

At low incident neutron energies, the INS reaction occurs predominantly through the compound nucleus mechanism, similar to fusion-evaporation reactions with charged particles. Therefore, the reaction leads to an alignment of the excited nuclei, so the γ-ray angular distributions from the decays of the excited levels exhibit anisotropies reflecting this alignment, the spins of the levels, and the multipolarities of the transitions. Typical angular distribution data, obtained with the ^112^Cd(n,n′ γ) reaction, are exhibited in Fig. 2.

## 4. Doppler-Shift Attenuation Measurements

**Fig. 3:**
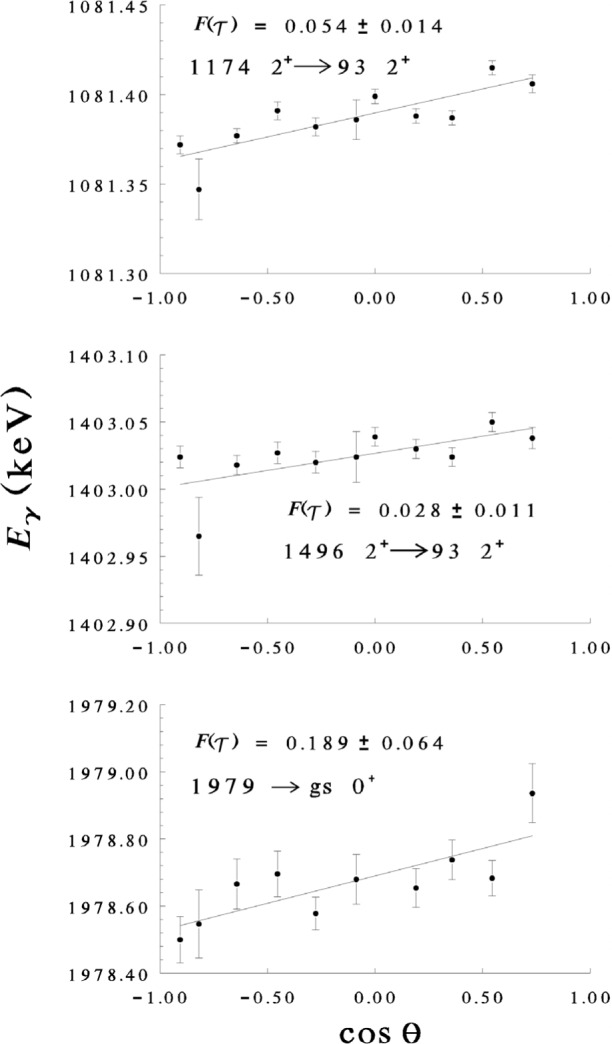
Plots of Doppler shifts for selected γ rays from the ^178^Hf(n,n′ γ) reaction using 2.0 MeV neutrons. The error bars reflect only the uncertainty on the *relative* change in energy at each angle; the absolute uncertainty on the energies are in general much greater. The extracted half lives for the levels at 1174 keV and 1496 keV are $0.42_{-0.09}^{+0.15}$ –0.09 ps and $0.8_{-0.2}^{+0.6}$ ps, respectively, compared to the previously measured values of 0.62 ps and 0.9 ps. The 1978 keV level (newly established) has a measured half life of $0.15_{-0.05}^{+0.09}$ ps.

A very exciting lifetime regime can be investigated in (n,n′ γ) measurements by employing the Doppler-shift attenuation method (DSAM). While the recoil velocity imparted (*v*/*c* ≃ 0.001) in neutron scattering reactions on heavy nuclei is small, it is sufficient to produce measurable Doppler shifts, and lifetimes in the fs to ps range can be determined. The Doppler-shifted γ-ray energy, *E*_γ_(θ_γ_), measured at a detector angle of θ_γ_ with respect to the incident neutrons can be related to *E*_0_, the energy of the γ ray emitted by a nucleus at rest, by the expression,

$$E_{\gamma }\left(\theta_{\gamma }\right)=E_{0}\left[1+F_{\exp}\left(\tau \right)\frac{v_{\mathrm{cm}}}{c}\;\cos\theta_{\gamma }\right]$$ (3)

where *v*_cm_ is the velocity of the center of mass in the inelastic neutron scattering collision with the nucleus, and *c* is the speed of light. *F*_exp_(τ) is the experimental attenuation factor determined from the measured Doppler shift and is compared with calculated attenuation factors to determine the lifetime. The formalism for determining nuclear lifetimes using DSAM methods with the (n,n′ γ) reaction have been described in detail [4]. Relative uncertainties of γ-ray energies for strong, well-resolved peaks can be determined to < 10 eV, so the largest source of uncertainty now resides in our lack of knowledge of the stopping powers of the recoiling residual nuclei. Figure 3 shows some typical Doppler shift curves obtained with the ^178^Hf(n,n′ γ) reaction with 2.0 MeV neutrons.

## 5. Gamma-Gamma Coincidence Measurements

For several years, we have performed γγ coincidence measurements using “beams” of fast neutrons produced with a collimation system developed in our laboratories. These measurements were initiated to permit the isotopic identification of coincident γ rays in experiments with samples of natural isotopic abundance; however, they proved so successful in providing new, unambiguous information that we now apply this method with isotopically enriched samples as well. The current four-detector “array” consists of the HPGe detectors in a close geometrical arrangement located as near as possible to the sample which is irradiated with collimated neutrons; the scattering sample and detector array are less than one meter from the source of neutrons. Much of this new methodology has been described in a manuscript providing details of our experimental facility [5].

## 6. Nuclear Structure Studies With the (n,n′γ) Reaction

The methods described above have been used to study the structure of a number of heavy nuclei. The nonselectivity of the INS reaction frequently enables the population of states that are inaccessible with other nuclear probes. Moreover, the DSAM measurements permit the determination of absolute transition probabilities, quantities of extreme value in examining the predictions of nuclear theories or in characterizing nuclear excitations.

Considerable progress has been made toward our understanding of multiphonon excitations in nuclei. The best evidence to date for the long-sought two-phonon octupole excitation in ^208^Pb has been obtained with the identification of a cascade of E3 transitions from a 0^+^ state at 5.241 MeV [6], and candidates for the other members of the quartet have been suggested [7]. In ^166^Er, both the K^π^ = 0^+^ and 4^+^ two-phonon γ-vibrational states have been uniquely identified [8]. Moreover, further study [9] of this nucleus has led to a characterization of all of the low-lying 0^+^ states, including a *β*-vibrational excitation near the two-phonon γ states, that is consistent with experimental data obtained with a variety of probes. Through lifetime measurements, the identification of three-phonon quadrupole excitations in ^112^Cd has been possible [10] and, for the first time, mixed-symmetry states in a good U(5) nucleus have been observed [11].

The role of dipole transitions at low excitation in nuclei has been a special focus of our work. A systematic examination of reduced E1 transition rates in the *N* = 82 region has been used to illustrate the ambiguities in determining the degree of nuclear collectivity from E1 strengths [12]. Other notable recent research accomplishments include the first observation of the two-phonon cascade of transitions from the (2^+^ ⊗ 3^−^) 1^−^ state of an *N* = 82 nucleus [13], thus confirming the two-phonon nature of this excitation, and the first direct lifetime measurements of M1 scissors mode excitations in deformed nuclei [14].

## 7. Conclusion

The inelastic neutron scattering reaction with γ-ray detection is complementary to other nuclear spectroscopic methods and has proven useful in exploring a number of features of the structure of stable nuclei at low angular momentum.
